# Validation of the Vision Impairment Screening Assessment in acute stroke care: a prospective diagnostic accuracy study

**DOI:** 10.1093/esj/aakag053

**Published:** 2026-05-29

**Authors:** Stephen James Ryan, Anna-Katharina E L Jørstad, Morten C Moe, Mona Skjelland, Ansar Roy, Fiona J Rowe, Øystein Kalsnes Jørstad, Anne Hege Aamodt

**Affiliations:** Department of Neurology, Oslo University Hospital, Oslo, Norway; Institute of Clinical Medicine, Faculty of Medicine, University of Oslo, Oslo, Norway; Department of Ophthalmology, Oslo University Hospital, Oslo, Norway; Institute of Clinical Medicine, Faculty of Medicine, University of Oslo, Oslo, Norway; Department of Ophthalmology, Oslo University Hospital, Oslo, Norway; Department of Neurology, Oslo University Hospital, Oslo, Norway; Institute of Clinical Medicine, Faculty of Medicine, University of Oslo, Oslo, Norway; Department of Neurology, Oslo University Hospital, Oslo, Norway; Institute of Population Health, Faculty of Health and Life Sciences, University of Liverpool, Liverpool, United Kingdom; Institute of Clinical Medicine, Faculty of Medicine, University of Oslo, Oslo, Norway; Department of Ophthalmology, Oslo University Hospital, Oslo, Norway; Department of Neurology, Oslo University Hospital, Oslo, Norway; Institute of Population Health, Faculty of Health and Life Sciences, University of Liverpool, Liverpool, United Kingdom; Department of Neuromedicine and Movement Science, The Norwegian University of Science and Technology, Trondheim, Norway

**Keywords:** vision impairment, stroke, screening, VISA, orthoptics, validation, NIHSS

## Abstract

**Introduction:**

Visual abnormalities are common after stroke but are often overlooked in acute care. The Vision Impairment Screening Assessment (VISA) was developed to support non-specialist clinicians in identifying visual abnormalities. This study evaluated the diagnostic accuracy of the Norwegian translation of VISA against structured neuro-ophthalmic examination in acute stroke. Secondary analyses examined the prevalence of early post-stroke visual abnormalities and compared VISA with the visual items of the National Institutes of Health Stroke Scale (NIHSS), the routine acute stroke assessment tool.

**Patients and methods:**

The Oslo Study for Vision Impairment after Stroke was a prospective diagnostic accuracy study including consecutive patients admitted for acute stroke management at a regional thrombectomy centre. Patients underwent bedside VISA assessment and neuro-ophthalmic examination within 72 h of admission. Diagnostic accuracy measures were calculated, and feasibility was assessed by completion rates and timing of assessments.

**Results:**

A total of 127 patients were included, of whom 81 completed both assessments. VISA demonstrated high sensitivity (94.5%) and lower specificity (34.6%), with a negative predictive value of 75.0%. Neuro-ophthalmic examination identified visual abnormalities in 70.1% of patients. The NIHSS visual items showed lower sensitivity (60.7%) than VISA, despite higher specificity (64.9%).

**Discussion and conclusions:**

Visual abnormalities were common in the acute phase after stroke and were more frequently detected by VISA than by the NIHSS, supporting routine early visual screening in acute stroke care beyond the NIHSS.

## Graphical Abstract

**Graphical Abstract:**
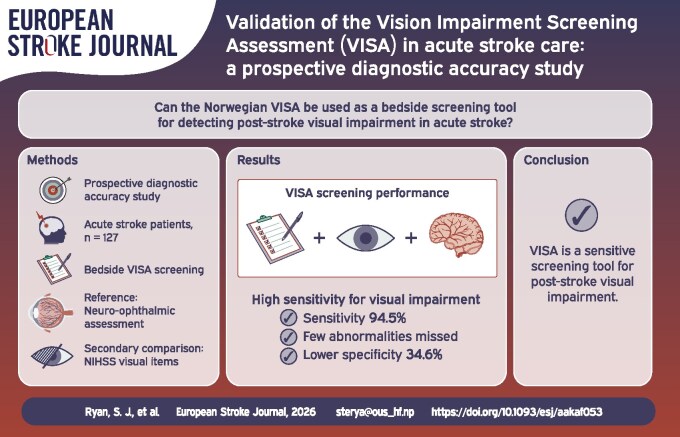

## Introduction

Vision is among the most complex and integrative functions of the human brain,^1^^,^^2^ relying on coordinated activity across widespread brain regions.^3^^,^^4^ Because cerebral ischaemia can affect any vascular territory, stroke has the potential to disrupt visual function at multiple levels, from pupillary responses and ocular motility to visual pathways and higher-order perceptual processes.^5^^,^^6^ As a result, visual abnormalities are a common but under-recognised consequence of stroke, with implications for patient safety, early mobilisation, rehabilitation planning and quality of life.^7–10^ Systematic investigations and guideline summaries estimate that 60%–75% of stroke survivors exhibit at least one measurable visual abnormality when formally assessed.^11^^,^^12^

Despite this high burden, structured assessment of vision in acute stroke care remains inconsistent.^13^ Screening for post-stroke visual abnormalities is recommended in international guidelines issued by major bodies, including the Intercollegiate Stroke Working Party, the National Institute for Health and Care Excellence and the European Stroke Organisation.^11^^,^^14^^,^^15^ These guidelines advise early structured screening using a validated tool or specialist eye-team assessment, delivered within routine acute care pathways.^11^ Recent national recommendations from the Norwegian Directorate of Health similarly advise systematic assessment of visual function using validated screening tools following acute stroke.^16^

Acute stroke assessment worldwide relies on the National Institutes of Health Stroke Scale (NIHSS), a standardised tool used to quantify stroke severity and support communication across the care pathway.^17–19^ Its visual items, Best Gaze, Visual Fields and Extinction or Inattention, assess horizontal gaze, hemianopic field loss and spatial neglect. Although the NIHSS includes visual components, it was not designed as a comprehensive assessment of visual function; its visual items primarily act as markers of neurological deficit rather than detailed evaluation of vision, as also highlighted in recent analyses of NIHSS item performance, and reliance on the NIHSS alone may therefore underestimate the spectrum of post-stroke visual abnormalities encountered in clinical practice.^20^^,^^21^ Consequently, several visual domains affected after stroke are not evaluated, including ocular motility beyond horizontal gaze, pupillary responses, reduced visual acuity and higher-order visual-perceptual dysfunction.^13^^,^^22^^,^^23^ Structured visual screening tools are therefore required to complement routine neurological assessment in acute stroke care.

The Vision Impairment Screening Assessment (VISA) was developed by the VISION research group at the University of Liverpool as a standardised, bedside screening method suitable for use by stroke clinicians without formal eye training.^24^ VISA was designed to evaluate the visual domains commonly affected by stroke: central vision, ocular alignment and motility, visual fields and visual attention.^25^

The primary aim of The Oslo Study for Vision Impairment after Stroke (StrokeVIS) was to validate the Norwegian translation of the VISA screening tool as a structured bedside screening tool to complement routine NIHSS-based stroke assessment. Diagnostic accuracy was evaluated against comprehensive neuro-ophthalmic examination as the reference standard for detecting visual abnormalities in early post-stroke patients admitted to a tertiary thrombectomy centre. A secondary aim was to quantify prevalence and characterise visual abnormalities in this setting. Despite strong guideline recommendations, validated vision screening tools are rarely implemented in hyperacute stroke care and evidence supporting feasibility and diagnostic performance in modern thrombectomy pathways remains limited.

## Patients and methods

### Study design and setting

StrokeVIS was a prospective, cross-sectional, single-centre diagnostic accuracy study, conducted at the Regional Stroke Unit, Oslo University Hospital, a tertiary-care comprehensive stroke centre providing endovascular therapy for multiple referring hospitals in South-Eastern Norway. This diagnostic accuracy analysis was conducted as part of the larger StrokeVIS clinical study investigating visual abnormalities after stroke. The study was reported in accordance with the Standards for Reporting of Diagnostic Accuracy Studies (STARD) guidelines for diagnostic accuracy studies.^26^

Patients were included between November 2021 and February 2024. Most were referred from other hospitals for evaluation for endovascular thrombectomy, resulting in rapid clinical turnover and heterogeneous stroke severity. This setting was selected to evaluate the practical feasibility of performing VISA screening in an acute stroke population undergoing hyperacute assessment and treatment.

### Ethics approval and consent to participate

The StrokeVIS study is registered at ClinicalTrials.gov NCT05809973. The study was approved by the Regional Committee for Medical and Health Research Ethics (reference no. 154964) and the Institutional Data Protection Officer at Oslo University Hospital (reference no. 20/19376) and was conducted in accordance with the Declaration of Helsinki. Written informed consent was obtained from all patients or legal representatives.

### Translation of the VISA tool

The Norwegian VISA translated version was developed using a structured forward–backward translation process. The original English tool was translated into Norwegian by a professional medical translator. A second independent translator subsequently back-translated the Norwegian version into English with input from a Norwegian orthoptist (AKELJ) to ensure appropriate discipline-specific terminology. The third stage compared the translated VISA and back-translated copy against the original English VISA. These versions were reviewed by a stroke neurologist at the Oslo University Hospital alongside discussion with the VISION Research Unit at the University of Liverpool, to ensure fidelity to the original instrument and confirm conceptual and linguistic accuracy. The final agreed translation was taken forward to clinical implementation.

### Participants

**Figure 1:**
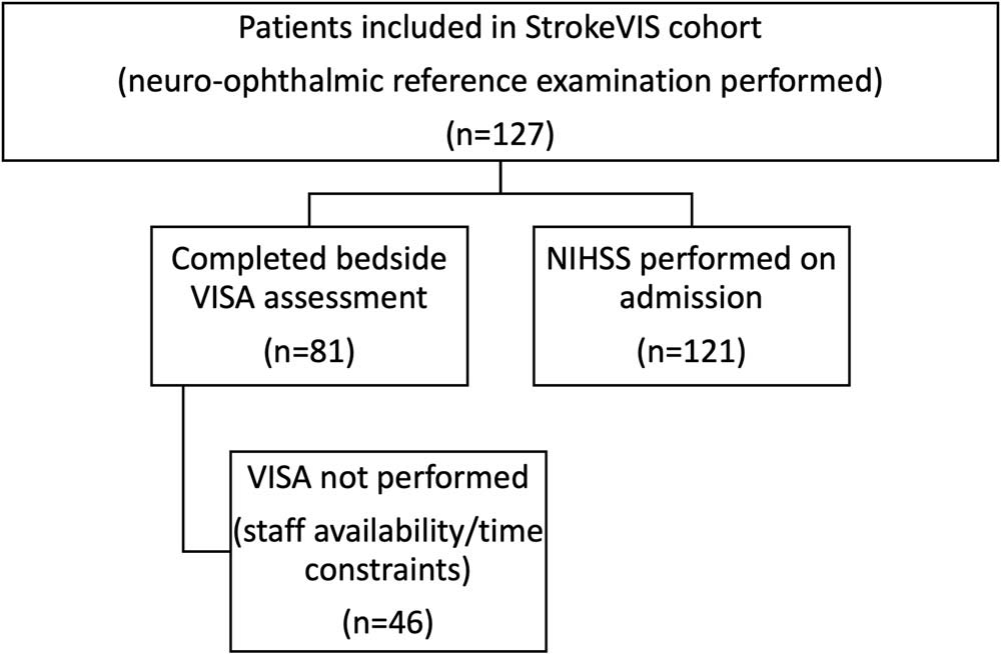
Participant flow and availability of Vision Impairment Screening Assessment (VISA), National Institutes of Health Stroke Scale (NIHSS) and neuro-ophthalmic assessments in the StrokeVIS study. Flow diagram showing the number of patients screened, included and completing each assessment (VISA, NIHSS and neuro-ophthalmic examination), with the diagnostic accuracy analysis based on patients who completed the reference standard neuro-ophthalmic examination.

Inclusion criteria were age ≥18, written informed consent by the patient or their legal representative, radiologically verified ischaemic or haemorrhagic stroke, ability to cooperate with the orthoptic examination and an admission NIHSS score of <20. Demographic and clinical variables were recorded, including sex, vascular risk factors (hypertension, diabetes, atrial fibrillation and smoking), stroke severity (NIHSS), functional status (modified Rankin Scale) and neuroimaging findings from computed tomography or magnetic resonance imaging. Participant recruitment and availability of VISA and NIHSS assessments are summarised in a STARD flow diagram (Figure 1).

### Neuro-ophthalmic assessment (reference standard)

All patients underwent a structured neuro-ophthalmic examination performed by an experienced orthoptist (AKELJ), which served as the reference standard. The assessment included a history of visual symptoms, evaluation of distance and near visual acuity, ocular alignment, binocular function and eye movements, including smooth pursuits and saccades. Eyelid and pupil responses were examined, and convergence was assessed. Visual inattention was evaluated using line bisection, clock drawing and symbol cancellation tasks, and visual fields were assessed by confrontation testing. Findings were categorised by visual acuity, ocular alignment and motility, visual fields and visual inattention to allow direct comparison with the VISA tool. Visual acuity worse than 0.2 logMAR (decimal visual acuity worse than 0.63) was considered a neuro-ophthalmological manifestation in participants with no prior history of visual acuity loss.^25^ All other neuro-ophthalmological manifestations were treated as categorical variables (present or absent).

### VISA screening (index test)

Patients also underwent structured screening using the Norwegian translation of the VISA, which was administered in paper format at the bedside by trained stroke nurses or physicians according to standardised instructions.^27^ In total, nine stroke nurses and one occupational therapist performed VISA assessments after receiving structured training in the use of the tool, including an instructional video, supervised practice and review of the standardised testing protocol.

### Blinding and timing of assessments

The VISA and neuro-ophthalmic examination were conducted within 72 h of admission and, where possible, within 24 h of each other to minimise potential for change in neuro-ophthalmic status between assessments. The order of assessments was determined by the availability of the orthoptist and patient. Examiners were blinded to the findings of the other assessment.

For comparison with routine acute stroke assessment, the visual components of the NIHSS—Best Gaze, Visual Fields and Extinction or Inattention—were extracted from the admission NIHSS recorded as part of standard clinical care.

For comparative analyses, distance visual acuity and other visual categories (ocular alignment/motility, visual fields and visual inattention) were included. Near visual acuity was excluded from comparative analyses because it could not be assessed consistently in the acute phase.

### Outcome measures

The primary outcome was diagnostic accuracy of the VISA screening tool in detecting visual abnormalities, compared with a structured neuro-ophthalmic examination, in acute stroke patients.

Secondary outcomes were the prevalence and distribution of visual abnormalities, the category-specific performance of VISA, comparison with NIHSS visual items, and the feasibility of VISA screening in acute stroke patients. Feasibility was defined as the proportion of eligible patients in whom VISA screening could be completed and the time interval between VISA screening and reference standard assessment.

### Statistical analyses

Analyses were performed using IBM SPSS Statistics, version 30.0. Continuous variables were summarised as mean and SD or median and interquartile range (IQR), as appropriate. Categorical variables were presented as counts and percentages.

Diagnostic accuracy of VISA was assessed using 2 × 2 contingency tables to calculate sensitivity, specificity, positive predictive value (PPV), negative predictive value (NPV) and overall accuracy, with 95% CI. Category-specific performance was evaluated similarly.

Agreement between the VISA and the NIHSS visual items was assessed using McNemar’s test. A 2-sided *P*-value <.05 was considered statistically significant. Feasibility was described using completion rates for VISA and the timing between assessments.

## Results

### Study population and feasibility

A total of 127 patients were included in the StrokeVIS cohort and underwent neuro-ophthalmic examination as the reference standard. Of these, 81 also completed bedside VISA screening and were included in the primary diagnostic accuracy analysis, while 121 had admission NIHSS data available for comparison with the reference standard; 77 completed all three assessments (Figure 1). Baseline demographic and clinical characteristics of the overall cohort are summarised in Table 1. The median interval between VISA and neuro-ophthalmic assessment was 0 days (IQR 0–1).

**Table 1:** Baseline characteristics of the study population.

Variable	Overall cohort *n* = 127
**Demographics**	
**Mean age, years (SD)**	65.5 (13.5)
**Men, n (%)**	75 (59.1)
**Stroke severity/function**	
**NIHSS on arrival^a^**	8 (3-14)
**NIHSS at discharge^b^**	2 (0-6)
**Pre-stroke mRS^c^**	
** 0**	102 (89.5)
** 1**	6 (5.3)
** 2**	2 (1.8)
** 3**	3 (2.6)
** 4**	1 (0.9)
** Missing**	13 (10.2)
**Acute therapy**	
**Intravenous thrombolysis (IVT), *n* (%)**	66 (52.0)
**Endovascular thrombectomy (EVT), *n* (%)**	78 (61.4)
**Combined IVT + EVT, *n* (%)**	42 (33.1)
**Comorbidities**	
**Atrial fibrillation, *n* (%)**	24 (18.9)
**Hypertension, *n* (%)**	58 (45.7)
**Hyperlipidaemia, *n* (%)**	61 (48.0)
**Diabetes mellitus, *n* (%)**	24 (18.9)
**Heart failure, *n* (%)**	16 (12.6)
**Previous stroke, *n* (%)**	26 (20.5)
**Hospital course**	
**Length of stay, days (SD)**	2.63 (1.89)

Baseline characteristics are presented for the full study cohort (*n* = 127). Subsets used for diagnostic accuracy analyses are described in the Results section and STARD flow diagram. Data are presented as number (%) unless otherwise specified. Continuous variables are presented as mean (SD) or median (interquartile range); minimum–maximum ranges are not shown. NIHSS values are presented as median (interquartile range).

Abbreviation: NIHSS = National Institutes of Health Stroke Scale.

^a^Available for 125 patients.

^b^Available for 117 patients.

^c^Available for 114 patients.

### Diagnostic accuracy of VISA

The primary diagnostic accuracy analysis included 81 patients who completed both VISA screening and the reference standard neuro-ophthalmic examination. VISA demonstrated high overall sensitivity (94.5%, 95% CI, 84.9–98.9) for detecting visual abnormalities, while specificity was lower (34.6%, 95% CI, 17.2–55.7). The PPV was 75.4% (95% CI, 63.5–84.9), and the NPV was 75.0% (95% CI, 42.8–94.5) (Table 2).

**Table 2:** Category-specific diagnostic accuracy of the Vision Impairment Screening Assessment (VISA) for detecting post-stroke visual abnormalities compared with neuro-ophthalmic examination.

Category	Sensitivity % (95% CI)	Specificity % (95% CI)	PPV % (95% CI)	NPV % (95% CI)	Accuracy^a^ %
**Overall** **(*n* = 81)**	94.5 (84.9–98.9)	34.6 (17.2–55.7)	75.4 (63.5–84.9)	75.0 (42.8–94.5)	75.3
**Distance visual acuity** **(*n* = 69)**	94.7 (74.0–99.9)	36.0 (22.9–50.8)	36.0 (22.9–50.8)	94.7 (74.0–99.9)	52.2
**Ocular motility** **(*n* = 81)**	54.8 (39.7–69.2)	76.9 (60.7–88.9)	71.9 (53.3–86.3)	61.2 (46.2–74.8)	65.4
**Visual field** **(*n* = 72)**	90.0 (55.5–99.7)	78.1 (66.0–87.5)	39.1 (19.7–61.5)	98.0 (89.6–100)	79.7
**Visual inattention** **(*n* = 72)**	88.9 (70.8–97.6)	68.9 (53.4–81.8)	63.2 (46.0–78.2)	91.2 (76.3–98.1)	76.4

Abbreviations: CI = confidence interval; NPV = negative predictive value; PPV = positive predictive value.

^a^Accuracy is the proportion of patients correctly classified by the VISA relative to the reference standard.

### Category-specific diagnostic accuracy of VISA

Category-specific diagnostic accuracy varied across visual domains (Table 2). Sensitivity was highest for distance visual acuity, visual field abnormalities and visual inattention, whereas ocular alignment and motility demonstrated lower sensitivity but comparatively higher specificity.

### Prevalence of visual abnormalities

**Figure 2:**
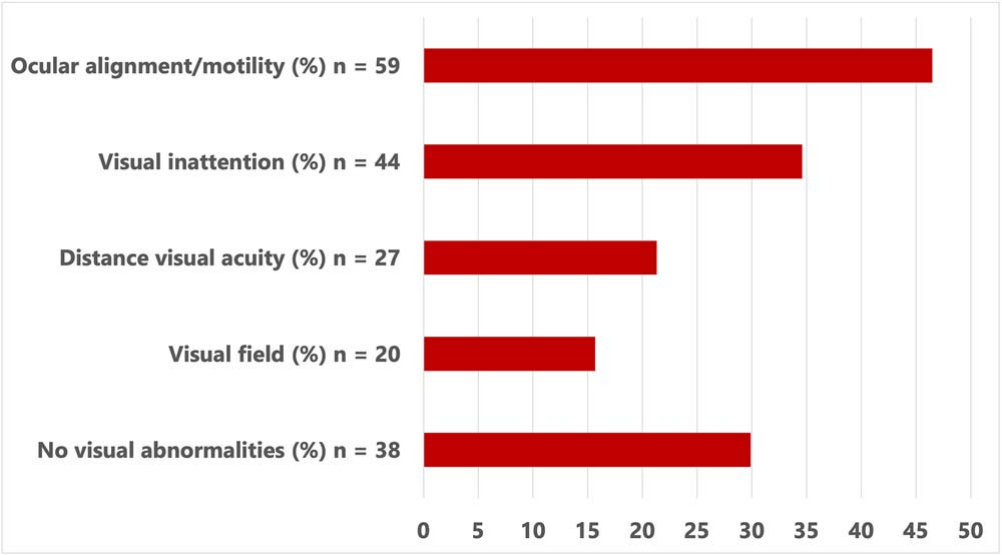
Prevalence of visual abnormalities detected by neuro-ophthalmic examination. Bars represent the proportion of patients (*n* = 127) with abnormalities in each visual domain. Percentages are calculated using the full study cohort as the denominator. Individual patients may have abnormalities in more than one domain. The category “no visual abnormalities” represents patients with no detected abnormalities. Some domains were not assessable in all patients in the acute setting.

Prevalence was assessed in all 127 enrolled patients. Visual abnormalities were defined as the presence of at least one abnormal finding on neuro-ophthalmic examination, with categorisation by ocular alignment/motility, visual fields, visual inattention and distance visual acuity. At least one visual abnormality was identified in 89 of 127 patients (70.1%). The prevalence of abnormalities within each visual domain is shown in Figure 2.

**Figure 3:**
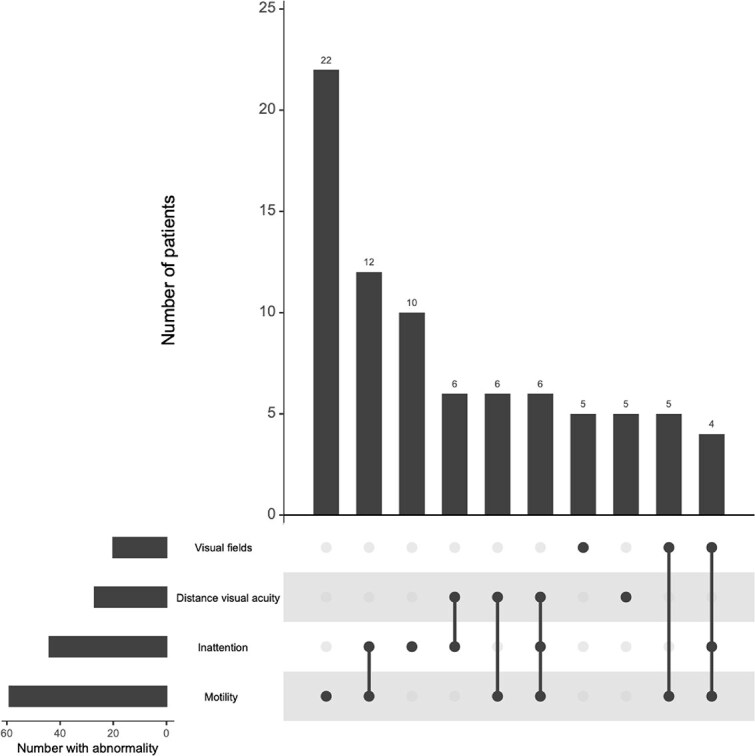
Co-occurrence of visual abnormalities detected by neuro-ophthalmic examination. UpSet plot illustrating overlap of visual abnormalities across ocular alignment/motility, visual fields, visual inattention and distance visual acuity in patients with complete data for these domains (*n* = 81). Bars indicate the number of patients with abnormalities in individual domains (set sizes) and the frequency of specific combinations of abnormalities (intersection sizes). Near visual acuity was not included in the co-occurrence analysis as it was not consistently assessed in all patients. The plot demonstrates the frequent co-occurrence of visual abnormalities across domains.

Visual abnormalities frequently co-occurred across domains. Co-occurrence patterns are illustrated in Figure 3, which includes patients with complete data across assessed domains (*n* = 81). This subset differs from the full cohort (*n* = 127), in which 89 patients had at least one abnormality. Near visual acuity was not included in the co-occurrence analysis due to incomplete assessment across the cohort.

### Comparison with NIHSS

For comparison with routine acute stroke assessment, the NIHSS visual items (Best Gaze, Visual Fields and Extinction or Inattention) were evaluated against the neuro-ophthalmic reference standard in the 121 patients with both assessments available. In this subgroup, the NIHSS visual items identified abnormalities in 64 patients and demonstrated lower sensitivity than VISA (60.7% vs 94.5%), but higher specificity (64.9% vs 34.6%) (Table 3).

**Table 3:** Overall diagnostic accuracy of the Vision Impairment Screening Assessment (VISA) and National Institutes of Health Stroke Scale (NIHSS) visual items compared with neuro-ophthalmic examination, and paired comparison of VISA versus NIHSS.

Measure	VISA *n* = 81	NIHSS *n* = 121
**Sensitivity**	94.5	60.7
**Specificity**	34.6	64.9
**Positive predictive value**	75.4	79.7
**Negative predictive value**	75.0	42.1
**Accuracy**	75.3	62.0
**McNemar test (VISA vs NIHSS)^a^**	χ^2^ = 20.8, *P* < .001	—

Values are percentages. Sensitivity and specificity are calculated relative to neuro-ophthalmic examination as the reference standard.

^a^McNemar test compares paired classification of visual abnormality (abnormal vs normal) between VISA and NIHSS in the 77 patients who completed both assessments. This test evaluates disagreement between the two tools and is not applicable to sensitivity or specificity estimates, which are calculated relative to the neuro-ophthalmic reference standard.

In the subset of 77 patients who completed VISA, NIHSS and neuro-ophthalmic examination, a paired comparison of overall abnormal versus normal classification demonstrated significant disagreement between VISA and the NIHSS visual items (McNemar’s χ^2^ = 20.8, *P* < .001) (Table 3). This test evaluates paired differences in classification between the two tools and does not apply to sensitivity or specificity estimates relative to the reference standard.

## Discussion

The StrokeVIS validation study demonstrates that the Norwegian translation of the VISA tool has high sensitivity for detecting post-stroke visual abnormalities when compared with comprehensive neuro-ophthalmic examination in an acute stroke population (sensitivity 94.5%). VISA identified nearly all patients with clinically confirmed abnormalities, supporting its use as a structured screening tool for early visual assessment in acute stroke care. At the same time, specificity was lower (34.6%), reflecting a pattern consistent with both the design of the tool and findings from prior validation studies.^25^ Together, these results indicate that VISA is well suited for identifying patients who require further specialist evaluation, while highlighting how clinical context and assessment conditions influence screening performance in acute settings.

Beyond detection alone, early identification of visual abnormalities is essential to ensure that patients are appropriately incorporated into treatment, follow-up and rehabilitation pathways. Unrecognised visual deficits may otherwise delay targeted interventions, contribute to functional impairment and increase the risk of complications such as falls, thereby adding to the overall burden of post-stroke disability.^28–30^

The observed diagnostic profile reflects the intended role of VISA as a screening instrument. As with screening tools generally, the VISA was designed to prioritise sensitivity over specificity, reflecting the established principle that screening aims to minimise false negatives and avoid missed clinically relevant abnormalities, with positive findings followed up by more detailed assessment.^26^^,^^31^ In this context, the negative predictive value was high, consistent with the high sensitivity observed, underscoring its suitability for early rule-out of clinically relevant visual abnormalities in acute stroke care.

Several factors likely contributed to reduced specificity in the present study. VISA was frequently administered in the acute phase, often shortly after thrombectomy or exposure to anaesthesia, when fatigue, reduced alertness, discomfort, or fluctuating attention may limit test performance. Assessor-related factors, including variation in test administration and interpretation, may contribute to reduced specificity, as previously described in structured vision screening studies.^32^ In addition, the lowest agreement between VISA and neuro-ophthalmic assessment was observed for distance visual acuity, resulting in a higher number of false-positive classifications and contributing disproportionately to reduced overall specificity. This finding mirrors observations from the original UK validation studies, where distance visual acuity screening was particularly sensitive to environmental factors such as suboptimal lighting, as well as patient-related factors including uncorrected refractive error and absence of appropriate spectacles in the acute setting.^25^ In this study, the tablet-based VISA application, which offers more controlled background illumination and has been shown to improve specificity for acuity testing, was not used.^25^ Ward-based screening with VISA should therefore consider either use of the app-based version or ensure adequate lighting conditions when paper-based testing is employed.

Accuracy varied across different visual domains. The VISA tool displayed higher accuracy for abnormalities that align closely with standard bedside evaluation methods, particularly visual field loss and visual inattention, where most findings confirmed on neuro-ophthalmic examination were detected. In contrast, ocular motility and distance visual acuity assessments diverged more from neuro-ophthalmic examination. These differences likely reflect variation in measurement characteristics between bedside screening and specialist neuro-ophthalmic assessment across different visual domains. Ocular motility assessment relies heavily on examiner observation and experience, whereas visual acuity testing is influenced by patient-related factors such as attention, cognition, fatigue and access to appropriate optical correction.^33^ When administered by stroke clinicians without specialist vision training, these factors may contribute differently to misclassification across test components. Importantly, across all components, VISA detected most true abnormalities, but patterns of sensitivity and specificity differed across visual domains.

From a clinical perspective, the high prevalence of visual abnormalities observed in this cohort has important implications for acute stroke care. Visual abnormalities were highly prevalent, affecting approximately 70% of patients. This high prevalence is consistent with previous reports and underscores the clinical relevance of systematic visual assessment in acute stroke management.^12^^,^^34^^,^^35^ Notably, the cohort was characterised by an acute, procedure-intensive context, including a high proportion of patients undergoing thrombectomy and rapid inter-hospital transfer. Such patients may be particularly challenging to screen, owing to fluctuating cognitive state, fatigue and competing clinical priorities, yet are also at substantial risk of unrecognised visual problems with implications for patient safety, including increased risk of falls and challenges during early rehabilitation.^33^

Feasibility, defined as the ability to complete structured visual assessment within the constraints of acute stroke care, was acceptable but imperfect. Most included patients underwent neuro-ophthalmic assessment within 72 h, and VISA, when performed, was typically administered within the same timeframe. However, VISA completion was incomplete, reflecting real-world constraints in a busy thrombectomy hub, including clinical instability, limited patient cooperation and early return to referring hospitals. These findings highlight the operational challenges of implementing relatively detailed screening tools in hyperacute environments and emphasise the importance of considering timing, workflow integration and testing conditions when introducing structured visual assessment into routine care.^12^^,^^35^

As expected, given its design, the visual items of the NIHSS substantially underestimated visual abnormalities in this cohort. Compared with the reference standard, the NIHSS demonstrated moderate specificity but markedly lower sensitivity, with approximately one-third of confirmed abnormalities remaining undetected. The negative predictive value was correspondingly low, indicating that a normal NIHSS visual score frequently occurred in patients with clinically relevant findings.

This reflects the intended scope of the NIHSS, which assesses horizontal gaze, visual fields and extinction but does not capture ocular motility beyond horizontal movements, visual acuity, or more subtle attentional and perceptual abnormalities.^22^^,^^23^^,^^36^ VISA identified more abnormalities than the NIHSS, consistent with the observed significant disagreement between the two assessments and reinforcing the added value of structured vision-specific screening.

These findings are consistent with previous studies demonstrating that dedicated vision-specific screening tools are required to identify the full spectrum of post-stroke visual abnormalities. Taken together, our findings support the European Stroke Organisation (ESO) guideline recommendation that structured vision screening should be incorporated into acute stroke pathways, even in hyperacute thrombectomy settings.^11^

The study has several strengths, including its prospective design, blinded assessments and use of a comprehensive specialist reference standard. Conducting the study within an acute thrombectomy pathway at the largest university hospital in Norway provides insights that are directly relevant to contemporary stroke services. The short interval between VISA and neuro-ophthalmic examinations, within 24 h, enhances internal validity by minimising the influence of neurological fluctuation and ensuring that both assessments reflect the same acute clinical state.

Several limitations should also be acknowledged. This was a single-centre study conducted at a tertiary thrombectomy hub, resulting in a cohort that was younger and more acutely treated than the general stroke population, which may limit generalisability to other settings. VISA completion was incomplete, introducing potential selection bias towards more clinically stable patients, which may have led to overestimation of both feasibility and diagnostic performance in routine acute stroke care. Near visual acuity was inconsistently assessable in the acute phase, largely due to patient fatigue, fluctuating alertness and frequent unavailability of appropriate near correction, which precluded direct comparison for this component. Inter-rater reliability was not examined, and the study was not designed to evaluate the impact of screening on downstream management, rehabilitation or functional outcomes. These considerations should inform interpretation of the findings and guide future implementation studies.

In conclusion, the Norwegian translation of VISA demonstrates high sensitivity for identifying post-stroke visual abnormalities in acute care, although specificity is reduced in hyperacute clinical contexts. Visual abnormalities are common in this population, and reliance on standard neurological scales such as the NIHSS alone risks under-recognition. Structured visual screening using VISA can enhance early detection, but its performance is influenced by testing environment, patient factors and workflow constraints. Taken together, these findings align with current ESO guideline recommendations that structured vision screening should form part of routine acute stroke pathways, including prior to discharge from acute stroke care.^11^ Routine use of VISA may improve early detection of visual abnormalities beyond standard neurological scales such as the NIHSS, supporting safer stroke care through earlier recognition of deficits with implications for mobilisation, rehabilitation and discharge planning.

## Data Availability

The datasets generated and/or analysed during the current study are available from the corresponding author upon reasonable request and subject to approval by relevant institutional and data protection authorities.
